# Genotypes of HLA, *TCF7L2*, and *FTO* as potential modifiers of the association between sweetened beverage consumption and risk of LADA and type 2 diabetes

**DOI:** 10.1007/s00394-019-01893-x

**Published:** 2019-01-17

**Authors:** Josefin E. Löfvenborg, Emma Ahlqvist, Lars Alfredsson, Tomas Andersson, Mozhgan Dorkhan, Leif Groop, Tiinamaija Tuomi, Alicja Wolk, Sofia Carlsson

**Affiliations:** 1 Institute of Environmental Medicine, Karolinska Institutet, Stockholm, Sweden; 2 Department of Clinical Sciences, Lund University, Malmö, Sweden; 3 Center for Occupational and Environmental Medicine, Stockholm County Council, Stockholm, Sweden; 4 Finnish Institute of Molecular Medicine, Helsinki University, Helsinki, Finland; 5 Division of Endocrinology, Abdominal Centre, Helsinki University Hospital, Helsinki, Finland; 6 Folkhälsan Research Center, Helsinki, Finland

**Keywords:** Autoimmune diabetes, Latent autoimmune diabetes in adults, T2D, Sweetened beverage, Genotype, BMI

## Abstract

**Purpose:**

Sweetened beverage consumption is associated with type 2 diabetes (T2D) and LADA. We investigated to what extent this association is mediated by BMI and whether it is modified by genotypes of HLA, *TCF7L2* rs7903146, or *FTO* rs9939609.

**Methods:**

Swedish case–control data including incident cases of LADA (*n* = 386) and T2D (*n* = 1253) with matched population-based controls (*n* = 1545) was used. We estimated adjusted ORs of diabetes (95% CI) in relation to sweetened beverage intake (per daily 200 mL serving) and genotypes. The impact of BMI was estimated using causal mediation methodology. Associations with HOMA-IR and HOMA-B were explored through linear regression.

**Results:**

Sweetened beverage intake was associated with increased risk of LADA (OR 1.15, 95% CI 1.03–1.29) and T2D (OR 1.21, 1.11–1.32). BMI was estimated to mediate 17% (LADA) and 56% (T2D) of the total risk. LADA was associated with risk variants of HLA (3.44, 2.63–4.50) and *TCF7L2* (1.27, 1.00–1.61) but not *FTO*. Only among non-carriers of high-risk HLA genotypes was sweetened beverage intake associated with risk of LADA (OR 1.32, 1.06–1.56) and HOMA-IR (beta = 0.162, *p* = 0.0047). T2D was associated with *TCF7L2* and *FTO* but not HLA, and the risk conferred by sweetened beverages appeared modified by *FTO* (OR 1.45, 95% CI 1.21–1.73 in non-carriers).

**Conclusions:**

Our findings suggest that sweetened beverages are associated with LADA and T2D partly through mediation by excess weight, but possibly also through other mechanisms including adverse effects on insulin sensitivity. These effects seem more pronounced in individuals without genetic susceptibility.

**Electronic supplementary material:**

The online version of this article (10.1007/s00394-019-01893-x) contains supplementary material, which is available to authorized users.

## Introduction

Sweetened beverage consumption has been associated with increased risk of type 2 diabetes (T2D) [1] and childhood type 1 diabetes (T1D) [2]. In a recent study based on Swedish data, we showed that sweetened beverage consumption may also increase the risk of latent autoimmune diabetes in adults (LADA) [3]. One proposed mechanism for its association with diabetes is that high consumption of sweetened beverages may lead to excess energy intake resulting in increased BMI [4], which in turn is a strong risk factor not only for T2D [5], but also for autoimmune diabetes [6, 7]. However, high BMI does not seem to fully explain the associations [1, 3]; there may also be direct effects on glucose metabolism including increased insulin resistance [8] and induced beta cell apoptosis [9].

Genetic susceptibility may modify the association between sweetened beverage intake and autoimmune diabetes. Findings in children suggest that consumption confers an elevated risk only among carriers of high-risk human leucocyte antigen (HLA) genotypes [2], which is the strongest genetic determinant of T1D [10]. LADA is a hybrid form of diabetes associated with HLA but also with transcription factor 7-like 2 (*TCF7L2*) [11], which confers the strongest genetic predisposition to T2D [12]. *TCF7L2* is involved in glucose homeostasis through the Wnt signaling pathway [13] and the minor T allele has been associated with impaired insulin secretion [14]. Furthermore, a recent meta-analysis found sweetened beverage intake to be associated with increases in fasting glucose and insulin [15]. Another gene of potential interest is the fat mass and obesity-associated (*FTO*) gene, which has been associated with overweight [16], possibly through alterations in satiety perception, energy intake [17], and nutrient preferences [18], but also associates with risk of T2D [19]. Sugar-sweetened beverages are likely to promote weight gain [20] and therefore it may be hypothesized that consumption would be more detrimental in terms of diabetes risk among individuals with genetic predisposition to overweight. To the best of our knowledge, only one previous study has investigated the possible interaction between sweetened beverage consumption and genetic susceptibility on the risk of T2D [21]. No evidence of interaction with a genetic risk score was found, but the role of individual genes was not investigated. Whether the association between sweetened beverages and autoimmune diabetes in adults is modified by genetic susceptibility has not been investigated previously.

Our aim was to clarify the influence of sweetened beverage consumption on LADA and T2D risk by investigating whether the association is modified by genotypes of HLA, *TCF7L2*, or *FTO*. These genes are suitable candidate genes for potential interaction with sweetened beverage intake due to their relatively strong genetic contribution to diabetes or overweight. We also aimed to explore underlying mechanisms and proportion of the observed association mediated by BMI.

## Subjects and methods

### Study population and design

This study is based on incident cases of LADA and T2D included in the ESTRID Study (Epidemiological Study of Risk Factors for LADA and Type 2 Diabetes, http://www.ki.se/imm/estrid), a Swedish case–control study ongoing since 2010 that has been described in detail elsewhere [22]. Cases were recruited from ANDIS (All New Diabetics in Scania, http://andis.ludc.med.lu.se), a diabetes registry aiming at including all new cases of diabetes identified and diagnosed by the health care providers within the county of Scania, and classify them based on clinical and genetic characteristics [23]. All incident cases of LADA and a random sample of T2D cases are invited to participate in ESTRID. Participation includes responding to an extensive questionnaire covering a wide range of health and lifestyle factors. The cases respond to the mailed questionnaire soon after diagnosis with 79% response rate. Controls in ESTRID are recruited from the Swedish Population Register through random selection and matched to the cases based on time and geographical region. These controls provide questionnaire data but no blood samples. For that reason, the present study instead uses data for diabetes-free controls from the EIRA Study (Epidemiological Investigation on Rheumatoid Arthritis, http://www.eirasweden.se), an ongoing, population-based case–control study utilizing similar methodology and questionnaire as ESTRID. The controls, randomly selected from the Swedish Population Register, were post-matched to the ESTRID cases by age and sex.

Eligible for the present study were all patients (*n* = 386 LADA, *n* = 1253 T2D) included in ESTRID until July 2017, and controls collected in EIRA 2005–2014 (*n* = 1545), aged ≥ 35 years and with complete information on lifestyle covariates and at least one of the three genetic factors. This study was approved by the Regional Ethical Review Board in Stockholm and all participants provided informed consent.

### Sweetened beverage and covariates

Habitual diet, as an average during the preceding year, was assessed using a validated [24–26] food-frequency questionnaire (FFQ). The FFQs were almost identical for ESTRID and EIRA and designed to cover usual diet. Patients with diabetes were specifically instructed to report their diet as it used to be prior to diagnosis. Sweetened beverages were assessed as the total intake of soft drinks/sodas, diluted syrups and nectars, but not 100% fruit juices. Consumption was reported as number of daily or weekly 200 mL servings. Validation of FFQ-reported sweetened beverage intake against four 1-week diet records in a subsample of 129 women showed a correlation of 0.6 (Wolk, unpublished results).

The ESTRID and EIRA questionnaires included identical questions on covariates. Self-reported height and weight were used to calculate BMI as weight in kilograms divided by the squared height in meters. Highest attained education was categorized as primary school, upper secondary school, or university. Leisure time physical activity during the preceding year (prior to diagnosis for cases) was reported as one of four response options ranging from sedentary to regularly active. Smoking habits of individuals were categorized as never smoker, former, or current smoker. Alcohol intake was estimated from the FFQ and categorized into none, 0.01–4.9 g/day, 5–14.9 g/day, and ≥ 15 g/day.

### Diabetes classification

Age at diagnosis, glutamic acid decarboxylase autoantibodies (GADA), and fasting C-peptide were used to determine diabetes subtype. Details of the serological assay methods have been described elsewhere [23]. GADA was measured with an enzyme linked immunosorbent assay (ELISA) with 84% sensitivity and 98% specificity at 10.7 IU/mL cut-off level [27]. Values above 250 IU/mL were censored at 250 IU/mL. Concentration of C-peptide was measured using Cobas e601 analyzer (Roche Diagnostics, Mannheim, Germany) or IMMULITE 2000 (Siemens Healthcare Diagnostics Product Ldt., Llanberis, UK). LADA was defined as age ≥ 35 years, GADA ≥ 10 IU/mL, and C-peptide ≥ 0.2 nmol/L (IMMULITE) or ≥ 0.3 nmol/L (Cobas e 601). Cases were classified as having T2D if age ≥ 35 years, GADA < 10 IU/mL, and C-peptide ≥ 0.60 nmol/L (IMMULITE) or ≥ 0.72 nmol/L (Cobas e 601). Homeostatic model assessment of insulin resistance (HOMA-IR) and beta cell function (HOMA-B) were calculated based on fasting plasma glucose and C-peptide [28].

### Genetic analyses

Blood samples for genotyping of patients were analyzed at the Clinical Research Center in Malmö, Sweden, using iPlex Gold Technology (Sequenom, San Diego, CA, USA). Missing genotypes were imputed for a subset using Infinium CoreExome v1.1 (Illumina, San Diego, CA, USA), based on the Haplotype Reference Consortium (http://www.haplotype-reference-consortium.org/; version r1.1 2016) reference panel. Controls were genotyped based on GWAS data generated through an Illumina Global Screening array or an Infinium Illumina 300K immunochip custom array (Illumina, San Diego, CA, USA). Genetic data used in the present study include three single nucleotide polymorphisms (SNPs) within the HLA gene region (rs3104413, rs2854275, rs9273363), one SNP within the *TCF7L2* gene (rs7903146), and one SNP within the *FTO* gene (rs9939609). HLA genotyping was done according to previously described methodology based on the three SNPs specified above, which have shown an accuracy of 99.3% [29]. Patients and controls were categorized as carriers of high-risk HLA genotypes: *DR4-DQ8, DR4*/*3-DQ8, DR3*/*4, DR3*/*3, DR4*/*4*, and DQA1*0501-DQB1*0201, or low/moderate-risk genotypes: *DR3*/*x, DR4*/*x, DR4-DQ7, DRx*/*x*, where x = neither *DR4* nor *DR3*. This categorization was based on the literature [30, 31] and frequency distributions in our study population. Participants were considered carriers of the risk variants of *TCF7L2* rs7903146 and *FTO* rs9939609 if they had at least one risk allele (i.e., genotypes TT/TC in *TCF7L2* rs7903146, and AA/AT in *FTO* rs9939609).

### Statistical analyses

Statistical Analysis Software (SAS) 9.4 (SAS Institute, Cary, NC, USA) was used for statistical analyses. Characteristics for patients and controls were presented as proportions, means, or medians (for skewed data), together with standard deviations (SD; for means) or interquartile range (IQR; for medians). Two-tailed *p* values were calculated by *χ*^2^ (proportions), Student’s *t* test (means), and Kruskal–Wallis *H* (medians) tests.

Conditional logistic regression was used to estimate odds ratios (OR) and 95% confidence intervals (CI) for the association between sweetened beverage consumption or genotype, and risk of LADA or T2D. Sweetened beverage consumption was analyzed in four categories and per one 200 mL daily serving increment in intake. Stratified analysis of the association between sweetened beverage intake and diabetes risk was done across genotypes of HLA (high or low/moderate risk), *TCF7L2* rs7903146 (TT/CT or CC) and *FTO* rs9939609 (AA/AT or TT). We also examined the presence of interaction, defined as departure from additivity of effects, between high sweetened beverage consumption (> 2 servings/day) and risk genotype by calculating attributable proportion due to interaction (AP). A significant positive interaction is indicated when AP > 0 and the confidence interval does not include 0 [32]. The change in log_e_ transformed HOMA-IR and HOMA-B, expressed as the regression coefficient, associated with one daily serving increase in sweetened beverages was assessed using linear regression.

All analyses were conditioned on age and sex (post-matching variables). Model 1 was adjusted for education, physical activity, smoking, and alcohol intake. Model 2 was also adjusted for BMI which was considered a mediator. Results from model 1 will be discussed in the text unless otherwise specified. Adjustment for dietary factors (red/processed meat, fatty fish, vegetables, fruit, sweet/salty snacks, and coffee) had little impact on the observed associations and were not included in the final models presented in this paper.

Using the approach suggested by VanderWeele [33], we assessed the mediating effect of BMI by estimating the natural direct and indirect effects, and proportion mediated, by means of the freely available SAS macro [34]. The method is designed to handle case–control data but does not account for the matched study design. This means that the estimates reflect the proportion of association mediated in our material, but hampers generalizability to the underlying population.

Sensitivity analysis included restricting analysis to controls from the years overlapping with the cases (i.e., 2010–2014), restricting the analysis to patients who filled out the questionnaire within 3 months of diagnosis, and restricting analysis to women only.

## Results

In comparison with T2D patients, LADA patients were younger, more often female, higher educated, more physically active, and had lower BMI (Table 1). The controls were leaner than the patients and the proportion of men was lower due to the fact that they originate from a matched study on rheumatoid arthritis, which predominantly affects women. This discrepancy in sex distribution is handled by post-matching. Patients with LADA were more often on insulin treatment and had worse beta cell function but lower degree of insulin resistance. Sweetened beverage intake did not differ between LADA and T2D patients (*p* = 0.9666), but was higher among cases compared to controls (0.59 vs. 0.28 servings/day, *p* < 0.0001).

**Table 1:** Characteristics of study participants

	Control	LADA	T2D	*p*^a^
No. of individuals	1545	386	1253	
Men, %	26.5	52.6	60.4	0.0064
Age (years), mean (SD)	57.6 (9.8)	58.6 (12.4)	63.2 (10.3)	< 0.0001
BMI (kg/m^2^), mean (SD)	25.4 (4.1)	27.9 (5.3)	31.1 (5.3)	< 0.0001
Sweetened beverage intake (serv/d), mean (SD)	0.28 (0.84)	0.59 (1.31)	0.59 (1.58)	0.9666
High education level, % university	36.6	28.2	20.4	0.0013
Low leisure time physical activity, %	10.9	17.4	23.3	0.0135
Current smoker, %	18.6	22.3	20.0	0.3393
Low alcohol intake (< 5 g/day), %	10.5 (12.8)	10.6 (19.8)	13.6 (42.4)	0.8066
High-risk HLA, %	31.6	61.2	31.4	< 0.0001
TT/TC in *TCF7L2* rs7903146, %	46.2	52.1	52.7	0.8225
AA/AT in *FTO* rs9939609, %	64.2	65.8	67.5	0.5561
With insulin treatment, %	–	47.0	5.6	< 0.0001
GADA (IU/mL), median (IQR)	–	240 (29–250)	–	–
HOMA-B, median (IQR)	–	32.8 (13.1–65.2)	68.1 (42.4–93.7)	< 0.0001
HOMA-IR, median (IQR)	–	2.70 (1.80–4.40)	3.60 (2.70–4.80)	0.0014

^a^*p* for the difference between LADA and type 2 diabetes

**Fig. 1:**
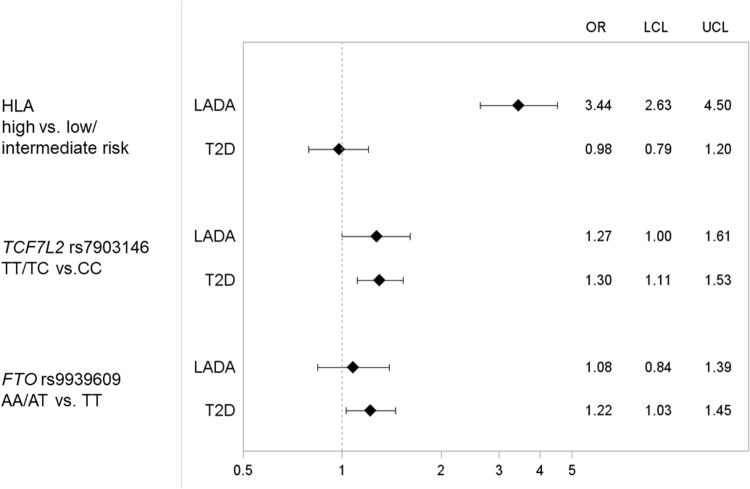
OR with 95% CI of incident LADA and type 2 diabetes in relation to genotypes of HLA, *TCF7L2*, and *FTO*. The model is adjusted for age and sex. Distribution of patients and controls with each genotype was as follows: LADA HLA low/intermediate risk *n* = 149, high risk *n* = 235; T2D HLA low/intermediate risk *n* = 851, high risk *n* = 389; controls HLA low/intermediate risk *n* = 601, high risk *n* = 278; LADA TCF7L2 CC *n* = 184, TT/TC *n* = 200; T2D TCF7L2 CC *n* = 587, TT/TC *n* = 655; controls TCF7L2 CC *n* = 823, TT/TC *n* = 707; LADA FTO TT *n* = 124, AA/AT *n* = 239; T2D FTO TT *n* = 392, AA/AT *n* = 814; controls FTO TT *n* = 548, AA/AT *n* = 983

**Fig. 2:**
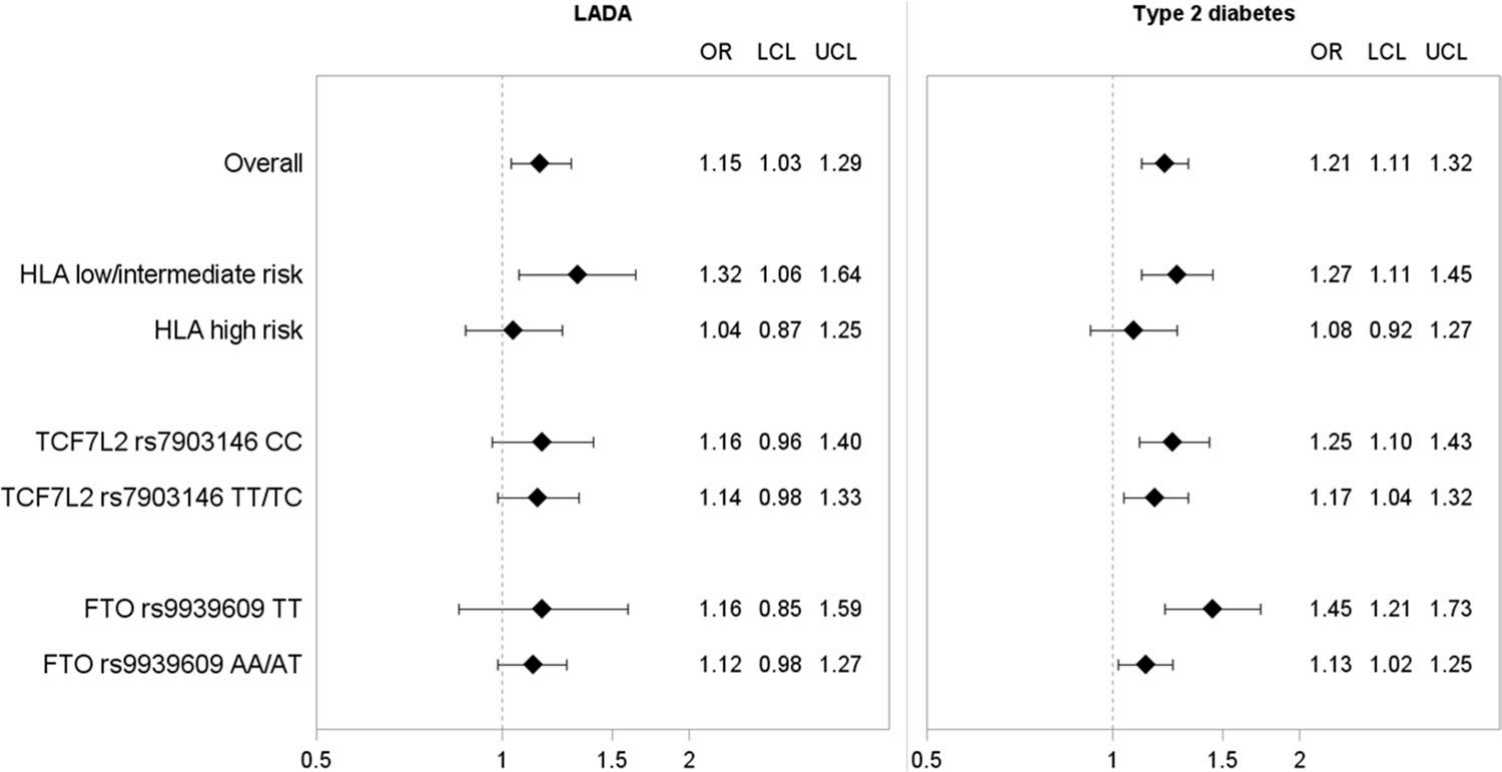
OR with 95% CI of incident LADA and type 2 diabetes per one daily serving of sweetened beverages by genotypes of HLA, *TCF7L2*, and *FTO*. The model is adjusted for age, sex, education, physical activity, smoking, and alcohol intake

LADA was associated with HLA and *TCF7L2* rs7903146 but not *FTO* rs9939609 (Fig. 1). Sweetened beverage intake was also associated with LADA: Each daily 200 mL serving increment corresponded to an OR of 1.15 (Fig. 2 and Supplementary Table 1). There was no evidence of interaction between sweetened beverages and any of the genotypes as indicated by AP, which means that the risk variants did not enhance the effect of sweetened beverage intake on diabetes risk (Supplementary Table 2). Instead, stratification by HLA genotype suggested that an increased risk of LADA in relation to sweetened beverage consumption was present primarily in carriers of low/intermediate risk genotypes. Further adjustment for BMI slightly attenuated the results (Supplementary Table 2). Neither *TCF7L2* rs7903146 nor *FTO* rs9939609 genotype seemed to modify the association between sweetened beverage intake and risk of LADA (Fig. 2 and Supplementary Table 3).

T2D was positively associated with genotypes of *TCF7L2* rs7903146 and *FTO* 9939609 but not HLA (Fig. 1). Sweetened beverage consumption was associated with 21% increased risk of T2D per one daily 200 mL serving increment (Fig. 2 and Supplementary Table 1). Similar to LADA, AP did not indicate any interaction between sweetened beverage intake and any of the genetic risk variants on the risk of T2D (Supplementary Table 2). Moreover, stratification by genotype showed no effect modification by *TCF7L2* (Fig. 2). However, the association between sweetened beverages and T2D was more pronounced in non-carriers of the risk variants of *FTO*, with minor influence of further BMI adjustment (Supplementary Table 1), and HLA (Fig. 2).

Insulin sensitivity increased by each daily serving of sweetened beverages in T2D patients (HOMA-IR beta = 0.035, *p* = 0.0007) (Supplementary Table 3). For LADA, such a relationship was apparent only among non-carriers of high-risk HLA genotypes (beta = 0.162, *p* = 0.0047). Additional adjustment for BMI had minor impact for both T2D and LADA. No changes in beta cell function related to sweetened beverage consumption were observed (Supplementary Table 3).

Categorical assessment of sweetened beverages suggested that consumption of more than two daily servings conferred an increased risk of both LADA (OR 2.04, 95% CI 1.11–3.77) and T2D (OR 3.34, 95% CI 2.11–5.28) compared to non-consumers (Supplementary Table 1). We subsequently used a mediation analysis approach and found BMI to mediate 17% of the association with LADA and 56% of the association with T2D among high consumers (Table 2). The natural direct effect of sweetened beverages on diabetes risk was estimated as a twofold increase in odds of both LADA and T2D.

**Table 2:** Estimates of direct effect and effect mediated through BMI of the association between sweetened beverage intake and LADA and T2D

> 2 serv/d vs. less	LADA	Type 2 diabetes
	OR (95% CI)^a^	OR (95% CI)^a^
Total effect	2.21 (1.22–3.99)	3.37 (1.78–6.35)
Natural direct effect	2.01 (1.07–3.76)	2.05 (1.22–3.43)
Natural indirect effect	1.10 (0.90–1.34)	1.64 (1.10–2.45)
Proportion mediated through BMI	17%	56%

^a^Model adjusted for age, sex, education, physical activity, smoking, and alcohol intake

The associations between LADA and T2D and one serving/day increase in sweetened beverage intake remained in the sensitivity analyses; restriction of controls from the years overlapping with the patients (i.e., 2010–2014) resulted in OR 1.18 (95% CI 1.00–1.38) for LADA and 1.28 (95% CI 1.12–1.47) for T2D, and restriction to patients who had responded to the questionnaire within 3 months of diagnosis showed OR 1.31 (95% CI 1.08–1.60) and OR 1.22 (95% CI 1.08–1.37) for LADA and T2D, respectively. OR from the analysis restricted to women, assessing the potential impact of the skewed sex distribution between patients and controls, was 1.20 (95% CI 1.02–1.41) for LADA and 1.37 (95% CI 1.20–1.56) for T2D per one daily serving.

## Discussion

Consumption of sweetened beverages is high worldwide and an important contributor to overweight [20] and also associated with diabetes risk [1]. Our findings indicate that sweetened beverage intake, in addition to its effect on overweight, may have a direct effect on the risk of both T2D and LADA and that this effect may be exerted through insulin resistance. Genetic risk variants did not seem to enhance the effect of sweetened beverage intake on diabetes risk, which concurs with the conclusions of a recent T2D study using a genetic risk score [21]. In contrast, consumption seemed to be a stronger risk factor among those with low genetic susceptibility for diabetes; in LADA, the highest risk conferred by sweetened beverages was seen in non-carriers of HLA risk genotypes whereas for T2D, the highest risk was seen among those not carrying the risk genotype of *FTO*. It is, however, important to note that the confidence intervals are overlapping and these findings thus need to be interpreted with caution.

In LADA, sweetened beverage intake was positively associated with insulin resistance only among those with low/intermediate-risk HLA genotypes. The pathogenesis of LADA is known to include both autoimmunity and insulin resistance, and the latter may be a promotor of less importance among individuals who are already at increased risk due to genetically induced autoimmunity. Our findings for LADA contradict previous findings for T1D in children, where sugar-sweetened beverage intake was positively associated with the progression from islet autoimmunity to clinical onset of diabetes only among high-risk HLA individuals [2]. There may be important differences in the pathophysiology between LADA and childhood T1D that could explain this discrepancy. Although the potential role of insulin resistance on T1D development has been brought to attention through the ‘accelerator hypothesis’ [35], it is likely to play a less pronounced role for childhood T1D [36] than for autoimmune diabetes with adult onset [37]. It has been proposed that a high sugar intake may be toxic to the beta cells leading to apoptosis [9] and that these detrimental effects may be exacerbated when autoantibodies are already present [38]. However, our findings did not indicate any changes in beta cell function. Of note, it has been reported that low/moderate-risk HLA genotypes have become more common among newly diagnosed T1D patients over the past decades, suggesting increased importance of environmental factors in the development of autoimmune diabetes [39]. We can also confirm that LADA is associated with *TC7FL2* [11], but not with *FTO* which is in contrast with a previous report [40]. Neither *TCF7L2* nor *FTO* seem to modify the association between sweetened beverages and risk of LADA.

To the best of our knowledge, this is the first study investigating whether the association between sweetened beverages and T2D is modified by *TCF7L2* and *FTO*. Our hypothesis was that the detrimental effect would be amplified in those with genetic susceptibility. Contrary to our hypothesis, there were no indications of synergistic effects; a positive association with sweetened beverage intake was observed across genotypes of both *TCF7L2* and *FTO*. In fact, the strongest association was found among those homozygous for the non-risk allele of *FTO*. One could speculate that carriers of the non-risk genotype of *FTO* have lower probability of becoming overweight and can therefore consume higher amounts of sweetened beverages without subsequent weight gain. If indeed sweetened beverages have detrimental effects on insulin resistance beyond what is mediated through BMI, this may explain our observed findings. In similarity with LADA, we found the association between sweetened beverages and T2D to be stronger in non-carriers of high-risk HLA genotypes. Importantly however, HLA genotype per se was not associated with T2D incidence, which is in concordance with the literature [41]. In this context, it is noteworthy that although GADA is the most frequent antibody found in LADA patients, 10% of patients are positive for other autoantibodies such as insulinoma-associated antigen-2 (IA-2A) or zinc transporter 8 (ZnT8A) [42]. Hence, it is possible that some of the patients classified as T2D, especially among carriers of HLA risk genotypes, are misclassified LADA patients and this could contribute to similarities in the results regarding the association with sweetened beverage intake. These findings should indeed be interpreted with caution; confirmations are clearly warranted.

Findings from the mediation analysis indicated that the direct effect of sweetened beverages on diabetes risk is of similar magnitude for LADA and T2D. This speaks in favor of a common underlying mechanism of equal importance. Beyond the direct effect, sweetened beverages seem to have an effect that is mediated through BMI which is more pronounced for T2D than for LADA. This is in line with a recent study indicating that overweight is a stronger risk factor for T2D than LADA [7]. Sweetened beverages were associated with insulin resistance also after adjustment for BMI, which suggests that it may be part of the observed direct effect. These findings confirm previous studies of both BMI as a mediator [1] and of insulin resistance as a possible driving force behind the observed associations [8].

The main strengths of our study are the population-based design and large number of incident LADA and T2D patients. We also have detailed dietary data from an extensively validated FFQ [24–26] as well as comprehensive information on important confounding factors. An important limitation is the retrospective nature of the collected data, which may lead to recall bias. However, the sensitivity analysis restricted to patients with the shortest diabetes duration indicates minor impact of such error. Furthermore, if patients have limited their sweetened beverage intake after diagnosis and reported accordingly, it would lead to underestimated ORs. In this context, it is important to note that our observed association between sweetened beverage intake and risk of T2D is in concordance with previous reports from prospective studies [1]. The controls were collected within a somewhat different context and time period and had a larger proportion of women than the cases. Sensitivity analysis indicated that this had minor influence on the results. Furthermore, the observed associations between sweetened beverage intake and diabetes correspond well to our previous findings based on the internal ESTRID controls with similar sex distribution and recruitment period [3]. Likewise, the association we observe for *TCF7L2*, and lack of association with HLA genotype, in relation to T2D risk is in line with previous findings [11]. Another important question is whether the sweetened beverage consumption of the controls reflects that of the population that generated the cases. In support hereof, we find that their mean consumption was quite similar to that of the general population according to the latest national survey by National Food Agency Sweden (in 2010, http://www.slv.se), slightly higher in male controls (0.47 vs. 0.42 servings/day) and slightly lower in female controls (0.21 vs. 0.28 servings/day). Importantly, mean consumption among the patients was higher than both the intake reported by controls and the reported average in the national survey. We could not distinguish between sugar-sweetened and artificially sweetened beverage, however, our previous findings indicate that the diabetes risk is increased irrespective of beverage type [3]. Neither was it possible to adjust for total energy intake and family history of diabetes, which are two potentially confounding factors, due to data availability. However, in our earlier publication using the same cases but controls collected within the ESTRID study [3], we found sweetened beverage intake to be positively associated with both LADA and T2D also after adjustment for these factors. Height and weight was self-reported for both cases and controls, which may lead to inaccurate BMI data. However, for the cases this shows high correlation with BMI based on clinical measurements from time of diagnosis (*r* = 0.92). Despite this, the use of BMI as a proxy for body fat is a crude measure [43]. Consequently, it is possible that we have underestimated the proportion of association mediated by BMI. Still, crude assessment of body fat mass is unlikely to explain the different results regarding mediation that we observed for LADA compared to T2D. The mediation analysis assumes a causal effect of sweetened beverages on both BMI and diabetes risk, which we are unable to prove.

In conclusion, these results indicate that high intakes of sweetened beverages increase the risk of both LADA and T2D, and that the effect is not amplified by genetic susceptibility conferred by genotypes of HLA, *FTO* or *TCF7L2*. The potential influence of sweetened beverages on diabetes risk seems to include effects on overweight as well as direct effects on insulin sensitivity. Thus, reducing sweetened beverage intake on the population level is likely to result in health gains both in terms of overweight and diabetes incidence.

## Electronic supplementary material

Below is the link to the electronic supplementary material.

Supplementary material 1 (DOCX 28 KB)
