# Supplementary material for: Genotypes of HLA, TCF7L2, and FTO as potential modifiers of the association between sweetened beverage consumption and risk of LADA and type 2 diabetes
Source: Eur J Nutr. 2019 Jan 17;59(1):127–35. doi: 10.1007/s00394-019-01893-x (PMC7000500; doi:10.1007/s00394-019-01893-x)
Supplement: Supplementary file 1 — Supplementary material 1 (DOCX 28 KB) [file 394_2019_1893_MOESM1_ESM.docx]

# SUPPLEMENTARY MATERIAL

# for manuscript “Genotypes of HLA, *TCF7L2*, and *FTO* as potential modifiers of the association between sweetened beverage consumption and risk of LADA and type 2 diabetes” by J.E. Löfvenborg et al.

Supplementary Table 1

Supplementary Table 2

Supplementary Table 3

**Supplementary Table 1.** Odds ratios (ORs) and 95% confidence intervals (CIs) of LADA and type 2 diabetes in relation to sweetened beverage intake in categories and per one daily serving increment, overall and by genotype of HLA (low/intermediate or high), *TCF7L2* rs7903146 (CC or TT/TC), and *FTO* rs9939609 (TT or AA/AT).

|  | | LADA | | | | |  |  | | Type 2 diabetes | | |  |
| --- | --- | --- | --- | --- | --- | --- | --- | --- | --- | --- | --- | --- | --- |
|  | | ca/co^a^ | OR (95% CI)^b^ | *p*^b^ | OR (95% CI)^c^ | *p*^c^ |  | ca/co^a^ | OR (95% CI)^b^ | | *p*^b^ | OR (95% CI)^c^ | *p*^c^ |
| Non-consumers | | 220/1104 | reference |  | reference |  |  | 798/1104 | reference | |  | reference |  |
| <1 serv/d | | 93/263 | 1.47 (1.07-2.03) | 0.0169 | 1.44 (1.04-1.98) | 0.0279 |  | 223/263 | 1.32 (1.04-1.68) | | 0.0216 | 1.13 (0.86-1.49) | 0.3903 |
| 1-2 serv/d | | 47/142 | 1.03 (0.69-1.53) | 0.9034 | 0.99 (0.66-1.49) | 0.9612 |  | 131/142 | 0.88 (0.66-1.19) | | 0.4145 | 0.77 (0.55-1.09) | 0.1418 |
| >2 serv/d | | 26/36 | 2.04 (1.11-3.77) | 0.0223 | 1.82 (0.97-3.43) | 0.0622 |  | 101/36 | 3.34 (2.11-5.28) | | <0.0001 | 2.08 (1.24-3.49) | 0.0058 |
| Per 1 daily serving | | 386/1545 | 1.15 (1.03-1.29) | 0.0170 | 1.12 (1.00-1.26) | 0.0599 |  | 1253/1545 | 1.21 (1.11-1.32) | | <0.0001 | 1.10 (1.00-1.21) | 0.0565 |
|  | |  |  |  |  |  |  |  |  | |  |  |  |
|  | HLA low/intermediate | 149/601 | 1.32 (1.06-1.64) | 0.0121 | 1.25 (1.00-1.56) | 0.0484 |  | 851/601 | 1.27 (1.11-1.45) | | 0.0007 | 1.13 (0.97-1.31) | 0.1161 |
|  | HLA high risk | 235/278 | 1.04 (0.87-1.25) | 0.6413 | 1.01 (0.84-1.21) | 0.9428 |  | 389/278 | 1.08 (0.92-1.27) | | 0.3554 | 0.92 (0.76-1.11) | 0.3795 |
|  |  |  |  |  |  |  |  |  |  | |  |  |  |
|  | *TCF7L2* rs7903146 CC | 184/823 | 1.16 (0.96-1.40) | 0.1200 | 1.13 (0.94-1.37) | 0.2042 |  | 587/823 | 1.25 (1.10-1.43) | | 0.0008 | 1.17 (1.01-1.35) | 0.0400 |
|  | *TCF7L2* rs7903146 TT/TC | 200/707 | 1.14 (0.98-1.33) | 0.0963 | 1.10 (0.94-1.29) | 0.2457 |  | 655/707 | 1.17 (1.04-1.32) | | 0.0089 | 1.05 (0.92-1.21) | 0.4685 |
|  |  |  |  |  |  |  |  |  |  | |  |  |  |
|  | *FTO* rs9939609 TT | 124/548 | 1.16 (0.85-1.59) | 0.3556 | 1.14 (0.82-1.57) | 0.4437 |  | 392/548 | 1.45 (1.21-1.73) | | <0.0001 | 1.41 (1.14-1.74) | 0.0014 |
|  | *FTO* rs9939609 AA/AT | 239/983 | 1.12 (0.98-1.27) | 0.0926 | 1.09 (0.96-1.25) | 0.1814 |  | 814/983 | 1.13 (1.02-1.25) | | 0.0155 | 1.02 (0.91-1.13) | 0.7658 |
| ^a^ ca/co = cases/controls | | | | | | | | | | | | |  |
| ^b^ Model 1 adjusted for age, sex, education, physical activity, smoking, and alcohol intake. | | | | | | | | | | | | |  |
| ^c^ Model 2 adjusted for same as model 1 + body mass index (BMI). | | | | | | | | |  | |  |  |  |

**Supplementary table 2.** Odds ratios (ORs) and 95% confidence intervals (CIs) of LADA and type 2 diabetes (T2D) for four mutually exclusive combinations of sweetened beverage intake and genotype. Attributable proportion due to interaction (defined as departure from additivity of effects) between high sweetened beverage consumption and risk genotype.

|  | | |  |  |  |  |  |  |  |  |  |  |  |  |
| --- | --- | --- | --- | --- | --- | --- | --- | --- | --- | --- | --- | --- | --- | --- |
| Type | | |  | HLA | | |  | *TCF7L2* | | |  | *FTO* | |  |
|  |  |  | Exposure combination^a^ | ca/co^b^ | OR (95% CI)^c^ | *p*^c^ |  | ca/co^b^ | OR (95% CI)^c^ | *p*^c^ |  | ca/co^b^ | OR (95% CI)^c^ | *p*^c^ |
| LADA | | |  |  |  |  |  |  |  |  |  |  |  |  |
|  | | | Low SB intake/low genetic risk | 137/587 | reference |  |  | 174/808 | reference |  |  | 119/539 | reference |  |
|  | | | Low SB intake/high genetic risk | 221/266 | 3.84 (2.89-5.10) | <0.0001 |  | 184/686 | 1.29 (1.01-1.66) | 0.0422 |  | 222/956 | 1.02 (0.78-1.32) | 0.8978 |
|  | | | High SB intake/low genetic risk | 12/14 | 2.89 (1.16-7.21) | 0.0225 |  | 10/15 | 1.72 (0.67-4.42) | 0.2591 |  | 5/9 | 1.49 (0.42-5.31) | 0.5364 |
|  | | | High SB intake/high genetic risk | 14/12 | 3.28 (1.32-8.12) | 0.0104 |  | 16/21 | 2.27 (1.05-4.93) | 0.0384 |  | 17/27 | 1.60 (0.77-3.34) | 0.2116 |
|  | | | Attributable proportion (95% CI) | -0.75 (-2.49; 1.00) | | |  | 0.11 (-0.86; 1.08) | | |  | 0.06 (-1.28; 1.39) | | |
| T2D | | |  |  |  |  |  |  |  |  |  |  |  |  |
|  | | | Low SB intake/low genetic risk | 784/587 | reference |  |  | 544/808 | reference |  |  | 355/539 | reference |  |
|  | | | Low SB intake/high genetic risk | 357/266 | 1.01 (0.81-1.26) | 0.9379 |  | 599/686 | 1.41 (1.18-1.68) | 0.0002 |  | 753/956 | 1.29 (1.06-1.56) | 0.0096 |
|  | | | High SB intake/low genetic risk | 67/14 | 3.52 (1.82-6.79) | 0.0002 |  | 43/15 | 3.27 (1.68-6.39) | 0.0005 |  | 37/9 | 6.37 (2.75-14.77) | <0.0001 |
|  | | | High SB intake/high genetic risk | 32/12 | 1.68 (0.79-3.58) | 0.1811 |  | 56/21 | 3.94 (2.17-7.18) | <0.0001 |  | 61/27 | 2.95 (1.71-5.09) | <0.0001 |
|  | | | Attributable proportion (95% CI) | -1.10 (-3.16; 0.96) | | |  | 0.07 (-0.70; 0.83) | | |  | -1.25 (-3.37; 0.86) | | |
|  | | |  |  |  |  |  |  |  |  |  |  |  |  |
|  |  | ^a^ Low sweetened beverage (SB) intake: ≤2 servings/day; High SB intake: >2 servings/day. | | | | | | | | | | | |  |
|  |  | ^b^ ca/co = cases/controls. | | | | | | | | | | | |  |
|  |  | ^c^ Model adjusted for age, sex, education, physical activity, smoking, and alcohol intake. | | | | | | | | | | | |  |
|  |  |  | | | | | | | | | | | |  |

| **Supplementary table 3.** Regression coefficient and p-value for the linear relationship between log_e_-transformed HOMA-IR and HOMA-B, respectively, and one daily serving increase in sweetened beverage intake among LADA and type 2 diabetes cases. | | | | | | | | | |  |
| --- | --- | --- | --- | --- | --- | --- | --- | --- | --- | --- |
|  |  |  |  |  |  |  |  |  |  |  |
|  | **LADA** | | | |  | **Type 2 diabetes** | | | | |
|  | HOMA-IR | | HOMA-B | |  | HOMA-IR | | HOMA-B | | |
| Per 1 daily serving | Beta^a^ (*p*) | Beta^b^ (*p*) | Beta^a^ (*p*) | Beta^b^ (*p*) |  | Beta^a^ (*p*) | Beta^b^ (*p*) | Beta^a^ (*p*) | Beta^b^ (*p*) | |
|  |  |  |  |  |  |  |  |  |  | |
| All | 0.051 (0.0882) | 0.043 (0.1406) | 0.015 (0.6986) | -0.006 (0.8561) |  | 0.035 (0.0007) | 0.033 (0.0014) | -0.016 (0.1708) | -0.019 (0.0989) | |
| **HLA** |  |  |  |  |  |  |  |  |  | |
| low/intermediate | 0.162 (0.0047) | 0.122 (0.0293) | 0.025 (0.7562) | -0.051 (0.5108) |  | 0.035 (0.0018) | 0.036 (0.0012) | -0.016 (0.2012) | -0.015 (0.2162) | |
| high risk | 0.008 (0.8165) | 0.005 (0.8958) | 0.013 (0.7601) | -0.001 (0.9819) |  | 0.037 (0.1260) | 0.021 (0.3881) | -0.006 (0.8032) | -0.027 (0.3025) | |
| ***TCF7L2*** |  |  |  |  |  |  |  |  |  | |
| CC | 0.090 (0.0753) | 0.082 (0.0976) | 0.085 (0.1641) | 0.068 (0.2374) |  | 0.033 (0.0102) | 0.034 (0.0075) | -0.026 (0.0684) | -0.025 (0.0788) | |
| TT/TC | 0.033 (0.3928) | 0.023 (0.5312) | -0.014 (0.7842) | -0.038 (0.3888) |  | 0.033 (0.0510) | 0.026 (0.1222) | 0.005 (0.7701) | -0.002 (0.9084) | |
| ***FTO*** |  |  |  |  |  |  |  |  |  | |
| TT | -0.013 (0.9153) | -0.015 (0.9019) | 0.025 (0.8667) | 0.018 (0.8976) |  | 0.020 (0.3083) | 0.019 (0.3438) | -0.027 (0.2343) | -0.029 (0.2033) | |
| AA/AT | 0.065 (0.0540) | 0.064 (0.0533) | -0.027 (0.5099) | -0.030 (0.4041) |  | 0.038 (0.0018) | 0.035 (0.0031) | -0.011 (0.4056) | -0.014 (0.2710) | |
|  |  |  |  |  |  |  |  |  |  | |
| ^a^ Change in HOMA (log_e_ transformed) per one additional daily serving of sweetened beverages; adjusted for age, sex, education, physical activity, smoking, and alcohol intake. | | | | | | | | | | |
| ^b^ Change in HOMA (log_e_ transformed) per one additional daily serving of sweetened beverages; adjusted for age, sex, education, physical activity, smoking, alcohol intake, and BMI. | | | | | | | | | |  |
